# Association between subtypes of metabolic syndrome and prognosis in patients with stage I endometrioid adenocarcinoma: A retrospective cohort study

**DOI:** 10.3389/fonc.2022.950589

**Published:** 2022-09-20

**Authors:** Man-qi Chen, Hai-xue Lin, Jin-xiao Liang, Miao-fang Wu, Jing Li, Li-juan Wang

**Affiliations:** ^1^ Department of Obstetrics and Gynecology, Sun Yat-sen Memorial Hospital, Sun Yat-sen University, Guangzhou, China; ^2^ Department of Obstetrics and Gynecology, The Second Affiliated Hospital of Hainan Medical University, Haikou, China; ^3^ Department of Gynecologic Oncology, Sun Yat-sen Memorial Hospital, Sun Yat-sen University, Guangzhou, China

**Keywords:** endometrioid adenocarcinoma, prognostic factor, hyperglycemia, metabolic syndrome, recurrence

## Abstract

**Purpose:**

To investigate the association between subtypes of metabolic syndrome (MetS) and prognosis of patients with stage I endometrioid adenocarcinoma.

**Patients and methods:**

Patients with stage I endometrioid adenocarcinoma who received surgical treatment as primary therapy at the Department of Gynecology of the Sun Yat-sen Memorial Hospital between June 2015 and December 2019 were retrospectively enrolled. According to the diagnosis criteria of MetS, the patients were categorized as patients without MetS, patients with MetS but without raised fasting plasma glucose (FPG, including previously diagnosed diabetes), and patients with MetS and raised FPG. All the included patients were followed from the dates of surgery until death, June 2021, or loss to follow-up, whichever came first, and cancer recurrence (including metastasis) was studied as the main outcome. Cox regression was used to evaluate the associations between subtypes of MetS and the study outcome adjusting for potential confounding factors.

**Results:**

Among the included 387 patients with stage I endometrioid adenocarcinoma, 193 (49.9%) were without MetS, 65 (16.8%) were with FPG not involving MetS, and 129 (33.3%) were with raised FPG involved MetS. With a median follow-up of 1,253 days, the cumulative incidence of cancer recurrence was 8.76% (95% confidence interval (CI) 2.5%–14.62%), 28.31% (95% CI 2.33%–47.38%), and 7.54% (95% CI 1.54%–13.17%), respectively. After adjusting for age, menopause, histological grade, tumor size, lymph-vascular space invasion, deep myometrial invasion, and treatments, comorbid FPG not involving MetS is a stronger risk factor of cancer recurrence than comorbid raised FPG involving MetS (hazard ratio 2.82 (95% CI 1.10–7.24) versus 1.18 (95% CI 0.45–3.13)) when compared to patients without MetS.

**Conclusion:**

Comorbid MetS generally presents as a risk factor of poor prognosis in patients with stage I endometrioid adenocarcinoma after surgical treatment, but the magnitude of the association may vary between subtypes, in which FPG not involving MetS appears to be predominant.

## Introduction

Uterine cancer is the second most common gynecologic cancer worldwide (following cervical cancer) but the most common one in resource-abundant countries, of which endometrial cancer accounts for more than 90% (1). Compared with other types of cancer in women, endometrial cancer is the fourth most common in the United States, with an incidence of 17–24 per 100,000 women (2). It has been observed that the incidence of endometrial cancer is increasing which may be related to the increase in relevant risk factors (including obesity and diabetes) (3). The prognosis of endometrial cancer differs by clinical and pathological features, and the 5-year overall survival rate ranges from 75% to 86% for patients with endometrioid endometrial cancer, while only 35% for patients with non-endometrioid endometrial cancer (4). With advances in diagnostic and therapeutic strategies, endometrial cancer is more and more common to be diagnosed at a relatively early stage and to receive appropriate treatment (5–8); however, the recurrence rate is still rather high [about 7% (9)] especially when considering that most patients were diagnosed at a relatively young age. It is therefore clinically relevant to identify risk factors of poor prognosis among early-staged endometrial cancer (10).

Metabolic syndrome (MetS) is a clustering of specific cardiovascular disease risk factors whose underlying pathophysiology is thought to be related to insulin resistance, which usually consists of several core components, including obesity, insulin resistance, dyslipidemia, and hypertension (11). MetS has been recognized as a risk factor of developing endometrial cancer in the past two decades, and the risk appears to increase with the increase with the number of MetS factors (12, 13). According to the traditional histomorphologic classification systems [by 1983, Bokhman (14)], type 1 endometrial cancer (i.e., mostly endometrioid histology) comprises the majority of endometrial cancer, which meanwhile usually occurs in younger women who are often obese or diabetic (15, 16). This makes MetS an important research topic of endometrioid carcinoma, and numerous studies examined the MetS and risk of developing endometrioid carcinoma (17, 18). However, only very few studies investigated the association between comorbid MetS and prognosis of patients who already developed endometrioid carcinoma (19–21). These available studies support comorbid MetS as a risk factor of poor prognosis, but it remains unclear whether subtypes of MetS modify the association, which had been investigated by only one study (21) as far as we know. Considering there are different diagnosis criteria of MetS, it is also necessary to confirm this association using other diagnosis criteria of MetS. Therefore, we performed a study to investigate the association between subtypes of MetS and prognosis of patients with stage I endometrioid adenocarcinoma. Specifically, we defined MetS according to the definition by the International Diabetes Federation (IDF) (22) and focused on subtypes according to whether the component-raised fasting plasma glucose (FPG, including previously diagnosed diabetes) was involved.

## Materials and methods

### Study population

Patients with stage I endometrioid adenocarcinoma who received surgical treatment as primary therapy at the Department of Gynecology of the Sun Yat-sen Memorial Hospital between June 2015 and December 2019 were retrospectively enrolled. In detail, a patient must meet all of the below inclusion criteria but none of the below exclusion criteria in order to be included. Inclusion criteria were (1) patients with stage I endometrial cancer according to the 2009 FIGO (International Federation of Gynecology and Obstetrics) staging system (23); (2) patients who received laparoscopic radical hysterectomy [class I or II, according to the Piver-Rutledge-Smith classification (24)] as primary treatment, without or with bilateral salpingo-oophorectomy, complete pelvic lymphadenectomy, and aortic lymph node dissection; (3) confirmed endometrioid adenocarcinoma based on histologic examination on surgical specimen. Exclusion criteria were (1) patients who received any other cancer treatment before the surgical treatment; (2) patients with other malignant tumors (synchronous or metachronous); (3) patients with other severe diseases (such as infection and organ failure) that might impact on survival; (4) the studied variables (see below) were unavailable.

The study was approved by the ethical committee of the Sun Yat-sen Memorial Hospital (No. SYSEC-KY-2022-125), and patient consents were waived because the study was a retrospective study and only routinely collected medical data were used. The study was conducted following the standards issued by the World Medical Association’s Declaration of Helsinki guidance.

### Exposures

We used the new IDF definition of MetS (22) to identify MetS, namely, central obesity, plus any two of the following factors: (1) raised serum triglycerides (≥1.7 mmol/l); (2) reduced serum high-density lipoprotein (HDL) cholesterol (<1.29 mmol/l); (3) raised blood pressure, defined as systolic blood pressure ≥130 mmHg, or diastolic blood pressure ≥85 mmHg, or previously diagnosed hypertension; (4) raised FPG (FPG ≥5.6 mmol/l), or previously diagnosed diabetes, which was identified by hemoglobin A1C (HbA1c) ≥6.5% in the current study.

According to the new IDF definition of MetS (22), central obesity should be defined as waist circumference ≥80 cm (for female Chinese), or body mass index (BMI) >30 kg/m^2^. However, in the study information about waist circumference was unavailable. We therefore followed the method suggested by Bozeman et al. (25) to develop a prediction model based on linear regression to predict waist circumference by age and BMI. Open supplemental data (licensed under a Creative Commons Attribution 4.0 International license, CC BY 4.0) including waist circumference, age, and BMI from 508 urban residents (164 men and 344 women) aged 19–70 years sampling from various districts of Shanghai, China, between 2012 and 2014 were obtained to develop the prediction model (26). After excluding women with missing values of waist circumference, age, or BMI, data of the remaining 312 women were used, of which the distributions and correlations (evaluated by Pearson correlation coefficient) were presented in **Supplemental Table 1** and **Supplemental Figure 1**. The model was developed by 10-fold cross-validation with linear regression, and the final model used to estimate the waist circumference of the patients with endometrioid adenocarcinoma in the current study was as follows: Waist circumference (cm) = 2.20730 × BMI (kg/m^2^) + 0.16377 × Age (years) + 22.23815. The multiple R-squared of this model was 0.6887, with a residual standard error of 5.131 on 309 degrees of freedom and an F-statistic of 341.7 on 2 and 309 degrees of freedom. A comparison of the predicted waist circumference by the final model to the true waist circumference of the datasets used for developing the model is also presented in **Supplemental Figure 2**.

According to the estimated waist circumference and the other factors of the diagnosis criteria of MetS (22), the patients were categorized as patients without MetS, patients with MetS but without raised fasting plasma glucose (FPG, including previously diagnosed diabetes), and patients with MetS and raised FPG. Therefore, the study exposure were subtypes of MetS, namely, without MetS, FPG not involving MetS, and raised FPG involving MetS.

### Outcomes

All the included patients were followed from the dates of surgery until death, June 2021, or loss to follow-up, whichever came first, and cancer recurrence (including metastasis) was studied as the main outcome and all-cause mortality was studied as the secondary outcome.

### Covariates

In addition to the variables mentioned above, namely, serum triglycerides, serum HDL cholesterol, systolic blood pressure, diastolic blood pressure, hypertension (history), FPG, HbA1c, age, BMI, and the estimated waist circumference, we also collected the below covariates: (1) menopause; (2) histological grade, categorized as low (well differentiated), moderate (moderately differentiated), and high (poorly differentiated); (3) (maximal) tumor size; (4) lymph-vascular space invasion; (5) deep myometrial invasion; (6) type of surgery [class I or II radical hysterectomy, according to the Piver-Rutledge-Smith classification (24)]; (7) adjuvant chemotherapy; (8) adjuvant radiotherapy.

### Statistical analysis

We present continuous variables as mean ± standard deviation or median (25th–75th percentile), and categorical variables as number and percentage. Cumulative incidences of the outcomes were estimated by the cumulative incidence competing risk (CICR) method or the Kaplan–Meier method for recurrence and all-cause mortality, respectively. Cox regression was used to evaluate the associations between subtypes of MetS and the study outcomes. We considered the below covariates as confounding factors which were adjusted in the regression model: age, menopause, histological grade, tumor size, lymph-vascular space invasion, deep myometrial invasion, type of surgery, adjuvant chemotherapy, and adjuvant radiotherapy. A two-sided P value less than 0.05 was considered as statistically significant. All the statistical analyses were performed by the R program (version 4.1.3, R Core Team (2020). R: A language and environment for statistical computing. R Foundation for Statistical Computing, Vienna, Austria).

## Results

### Baseline characteristics of the included patients

**Figure 1:**
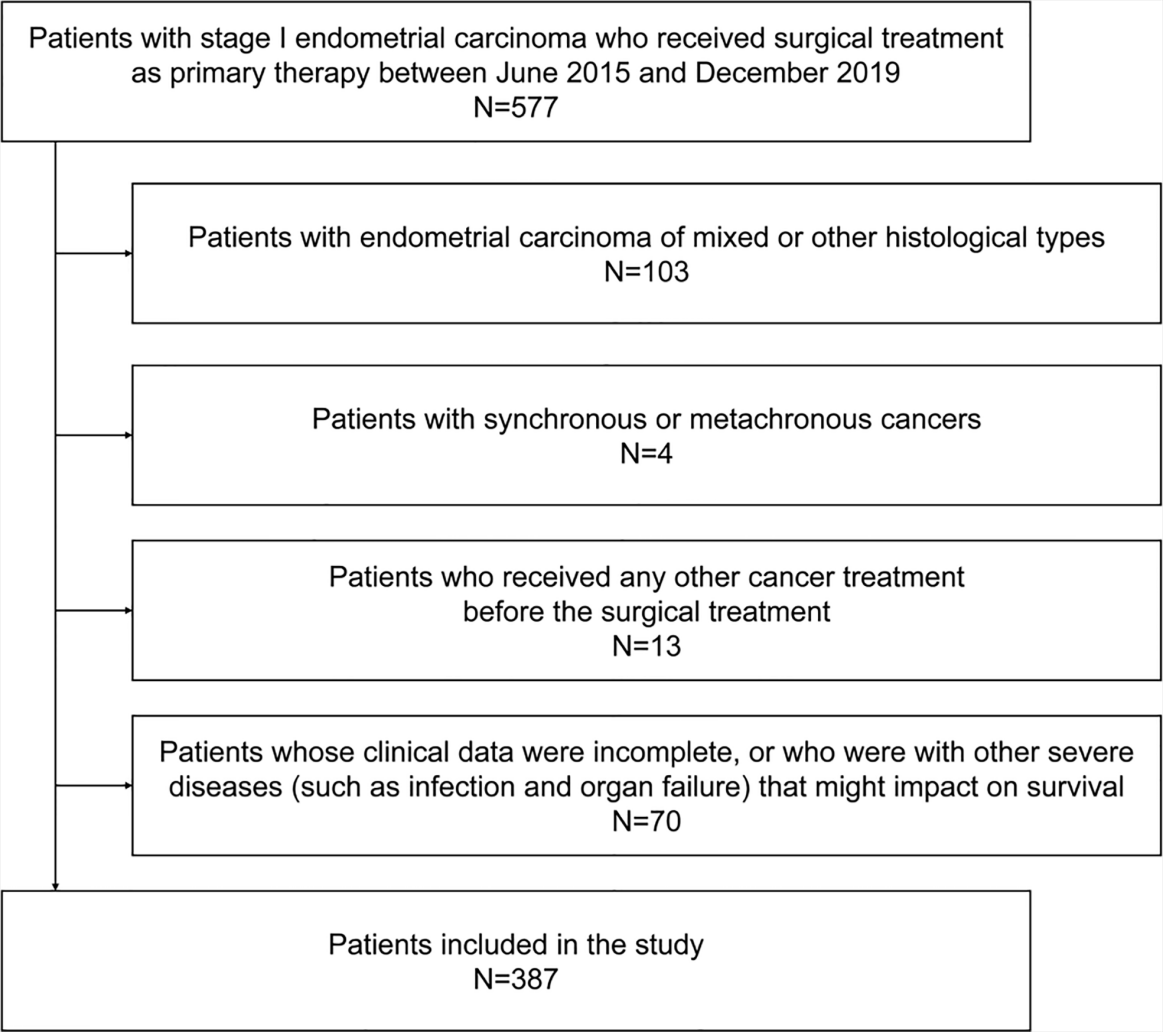
Flow graph for the inclusion of the study population.

A total of 387 patients with stage I endometrioid adenocarcinoma who received surgical treatment as primary therapy were included (**Figure 1**). The mean age was 52.5 ± 8.3 years, and 48.6% experienced menopause. The mean BMI of the patients was 24.6 ± 3.8 kg/m^2^, of which 15.8% were ≥28 kg/m^2^. The mean (estimated) waist circumference was 85.1 ± 8.6 cm, of which 70.3% ≥80 cm met the definition of central obesity. Among the patients, only 10.3% were with a high histological grade, and the median (maximal) tumor size was 2.0 cm (25th–75th percentiles 0.5–3.4 cm). Lymph-vascular space invasion and deep myometrial invasion were found in 17.8% and 12.9% of the patients, respectively. The majority (89.4%) of the patients received class I radical hysterectomy, 10.6% received adjuvant chemotherapy, and 17.3% received adjuvant radiotherapy. Other baseline characteristics are found in **Table 1**.

**Table 1:** Patient characteristics at baseline.

Patient characteristics	All patients (n = 387)
Age (years)	52.5 ± 8.3
Menopause	188 (48.6%)
BMI (kg/m^2^)	24.6 ± 3.8
<18.5	18 (4.7%)
18.5-23.9	165 (42.6%)
24-27.9	143 (37.0%)
≥28	61 (15.8%)
Waist circumference (cm)^†^	85.1 ± 8.6
≥80 cm	272 (70.3%)
Triglycerides (serum, mmol/L)	1.8 ± 1.2
≥1.7 mmol/l	144 (37.2%)
HDL cholesterol (serum, mmol/L)	1.2 ± 0.3
<1.29 mmol/l	231 (59.7%)
Systolic blood pressure (mmHg)	129.5 ± 18.2
≥130 mmHg	180 (46.5%)
Diastolic blood pressure (mmHg)	80.0 ± 9.7
≥85 mmHg	114 (29.5%)
Hypertension (history)	155 (40.1%)
HbA1c (%)	5.7 ± 1.1
HbA1c ≥6.5%	52 (13.4%)
Fasting glucose (plasma, mmol/L)	5.7 ± 1.7
≥5.6 mmol/l	159 (41.1%)
Histological grade	
Low (well differentiated)	192 (49.6%)
Moderate (moderately differentiated)	155 (40.1%)
High (poorly differentiated)	40 (10.3%)
Tumor size (cm)	2.0 (0.5-3.4)
Lymph-vascular space invasion	69 (17.8%)
Deep myometrial invasion	50 (12.9%)
Type of surgery (Piver–Rutledge classification)	
Class I	346 (89.4%)
Class II	41 (10.6%)
Adjuvant chemotherapy	41 (10.6%)
Adjuvant radiotherapy	67 (17.3%)

†Estimated by the formula: Waist circumference (cm) = 2.20730 × BMI (kg/m^2^) + 0.16377 × Age (years) + 22.23815.

BMI, body mass index; HDL, high-density lipoprotein; HbA1c, hemoglobin A1C.

### MetS in the included patients

Among the included 387 patients, 194 (50.1%) met the criteria of MetS. Among the patients without MetS, 40.4% were with central obesity, 15.0% were with raised triglycerides, 34.2% were with reduced HDL cholesterol, 36.3% were with raised blood pressure, and 16.6% were with raised FPG. Among the patients with MetS, the prevalence of reduced HDL cholesterol and raised blood pressure was both about 80%, while only about 60% had raised triglycerides or raised FPG (**Table 2**). All the four factors (n = 51), reduced HDL cholesterol plus raised blood pressure (n = 30), and reduced HDL cholesterol plus raised blood pressure plus raised FPG (n = 26) were the three most frequent patterns that met the diagnosis criteria of MetS (**Supplemental Table 2**). As presented in **Supplemental Table 3**, among the four factors of the diagnosis criteria of MetS, raised triglycerides were more often to present with reduced HDL cholesterol (84.3%), while reduced HDL cholesterol was more often to present with raised blood pressure (78.8%) and vice versa.

**Table 2:** Patient characteristics at baseline according to metabolic syndrome diagnosed by the new International Diabetes Federation (IDF) definition.

Patient characteristics	All patients (n = 387)	Without metabolic syndrome (n = 193)	Metabolic syndrome (n = 194)
Central obesity	272 (70.3%)	78 (40.4%)	194 (100%)
Waist circumference^†^ ≥80 cm	272 (70.3%)	78 (40.4%)	194 (100%)
BMI >30 kg/m^2^	35 (9.0%)	8 (4.1%)	27 (13.9%)
Raised triglycerides (≥1.7 mmol/l, serum)	144 (37.2%)	29 (15.0%)	115 (59.3%)
Reduced HDL cholesterol (<1.29 mmol/l, serum)	231 (59.7%)	66 (34.2%)	165 (85.1%)
Raised blood pressure	226 (58.4%)	70 (36.3%)	156 (80.4%)
Systolic blood pressure ≥130 mmHg	180 (46.5%)	60 (31.1%)	120 (61.9%)
Diastolic blood pressure ≥85 mmHg	114 (29.5%)	39 (20.2%)	75 (38.7%)
Hypertension (history)	155 (40.1%)	35 (18.1%)	120 (61.9%)
Raised FPG	161 (41.6%)	32 (16.6%)	129 (66.5%)
FPG ≥5.6 mmol/l	159 (41.1%)	31 (16.1%)	128 (66.0%)
HbA1c ≥6.5%	52 (13.4%)	6 (3.1%)	46 (23.7%)
Number of the four factors of metabolic syndrome^‡^			
0	58 (15.0%)	58 (30.1%)	–
1	94 (24.3%)	94 (48.7%)	–
2	92 (23.8%)	24 (12.4%)	68 (35.1%)
3	88 (22.7%)	13 (6.7%)	75 (38.7%)
4	55 (14.2%)	4 (2.1%)	51 (26.3%)

†Estimated by the formula: Waist circumference (cm) = 2.20730 × BMI (kg/m^2^) + 0.16377 × Age (years) + 22.23815.

‡Raised triglycerides, reduced HDL cholesterol, raised blood pressure, and raised FPG.

BMI, body mass index; HDL, high-density lipoprotein; FPG, fasting plasma glucose; HbA1c, hemoglobin A1C.

When categized as three groups, among the total 387 patients, 193 (49.9%) were without MetS, 65 (16.8%) were with FPG not involving MetS, and 129 (33.3%) were with raised FPG involving MetS. Compared to patients without MetS, except for difference in the factors that consisted the diagnosis criteria of MetS, the other two groups of patients were older, with higher proportions of menopause, high (poorly differentiated) histological grade, lymph-vascular space invasion, deep myometrial invasion, and receiving adjuvant chemotherapy or radiotherapy (**Table 3**).

**Table 3:** Patient characteristics at baseline according to subtypes of metabolic syndrome.

Patient characteristics	Without MetS (n = 193)	FPG not involving MetS^†^ (n = 65)	Raised FPG involving MetS^†^ (n = 129)
Age (years)	51.5 ± 8.8	53.5 ± 9.3	53.4 ± 6.9
Menopause	83 (43.0%)	32 (49.2%)	73 (56.6%)
BMI (kg/m^2^)	22.5 ± 3.2	26.3 ± 3.0	26.9 ± 3.3
Waist circumference (cm)^‡^	80.3 ± 7.3	89.0 ± 6.1	90.3 ± 7.4
Triglycerides (serum, mmol/L)	1.4 ± 1.3	2.1 ± 1.1	2.2 ± 0.9
HDL cholesterol (serum, mmol/L)	1.3 ± 0.3	1.1 ± 0.2	1.0 ± 0.3
Systolic blood pressure (mmHg)	123.3 ± 16.6	137.2 ± 15.0	135.0 ± 18.9
Diastolic blood pressure (mmHg)	77.8 ± 9.5	84.7 ± 9.9	81.0 ± 8.9
Hypertension (history)	35 (18.1%)	40 (61.5%)	80 (62.0%)
HbA1c (%)	5.3 ± 0.7	5.3 ± 0.5	6.5 ± 1.4
Fasting glucose (plasma, mmol/L)	5.0 ± 0.8	4.8 ± 0.5	7.3 ± 2.1
Histological grade			
Low (well differentiated)	96 (49.7%)	32 (49.2%)	64 (49.6%)
Moderate (moderately differentiated)	81 (42%)	22 (33.8%)	52 (40.3%)
High (poorly differentiated)	16 (8.3%)	11 (16.9%)	13 (10.1%)
Tumor size (cm)	1.5 (0.5-3.0)	2.3 (0.6-3.6)	2.0 (1.0-3.5)
Lymph-vascular space invasion	21 (10.9%)	14 (21.5%)	34 (26.4%)
Deep myometrial invasion	23 (11.9%)	11 (16.9%)	16 (12.4%)
Type of surgery (Piver–Rutledge classification)			
Class I	176 (91.2%)	58 (89.2%)	112 (86.8%)
Class II	17 (8.8%)	7 (10.8%)	17 (13.2%)
Adjuvant chemotherapy	15 (7.8%)	8 (12.3%)	18 (14%)
Adjuvant radiotherapy	30 (15.5%)	14 (21.5%)	23 (17.8%)
Central obesity	78 (40.4%)	65 (100%)	129 (100%)
Raised triglycerides	29 (15.0%)	35 (53.8%)	80 (62.0%)
Reduced HDL cholesterol	66 (34.2%)	60 (92.3%)	105 (81.4%)
Raised blood pressure	70 (36.3%)	58 (89.2%)	98 (76.0%)
Raised FPG	32 (16.6%)	0 (0%)	129 (100%)
Number of the four factors of MetS^*^			
0	58 (30.1%)	0 (0%)	0 (0%)
1	94 (48.7%)	0 (0%)	0 (0%)
2	24 (12.4%)	42 (64.6%)	26 (20.2%)
3	13 (6.7%)	23 (35.4%)	52 (40.3%)
4	4 (2.1%)	0 (0%)	51 (39.5%)

†Raised FPG involved MetS refers to MetS diagnosed according to the new International Diabetes Federation (IDF) definition and the patients were with the factor “raised FPG” which were determined by FPG ≥5.6 mmol/l and/or HbA1c ≥6.5%. The rest of the MetS patients were categorized as FPG not involved MetS.

‡Estimated by the formula: Waist circumference (cm) = 2.20730 × BMI (kg/m^2^) + 0.16377 × Age (years) + 22.23815.

*Raised triglycerides, reduced HDL cholesterol, raised blood pressure, and raised fasting plasma glucose.

MetS, metabolic syndrome; FPG, fasting plasma glucose; BMI, body mass index; HDL, high-density lipoprotein; HbA1c, hemoglobin A1C.

When compared to patients with raised FPG involving MetS, although patients with FPG not involving MetS had similar ages and similar distributions of the factors that consisted the diagnosis criteria of MetS (except for raised FPG and HbA1c), they had fewer numbers of the four factors (proportion of having only two factors: 64.6% versus 20.2%). In addition, patients with FPG not involving MetS had a lower proportion of menopause, lymph-vascular space invasion, and receiving adjuvant therapy but a higher proportion of high histological grade and deep myometrial invasion when compared to those with raised FPG involving MetS (**Table 3**.)

### Associations of subtypes of MetS with prognosis

**Figure 2:**
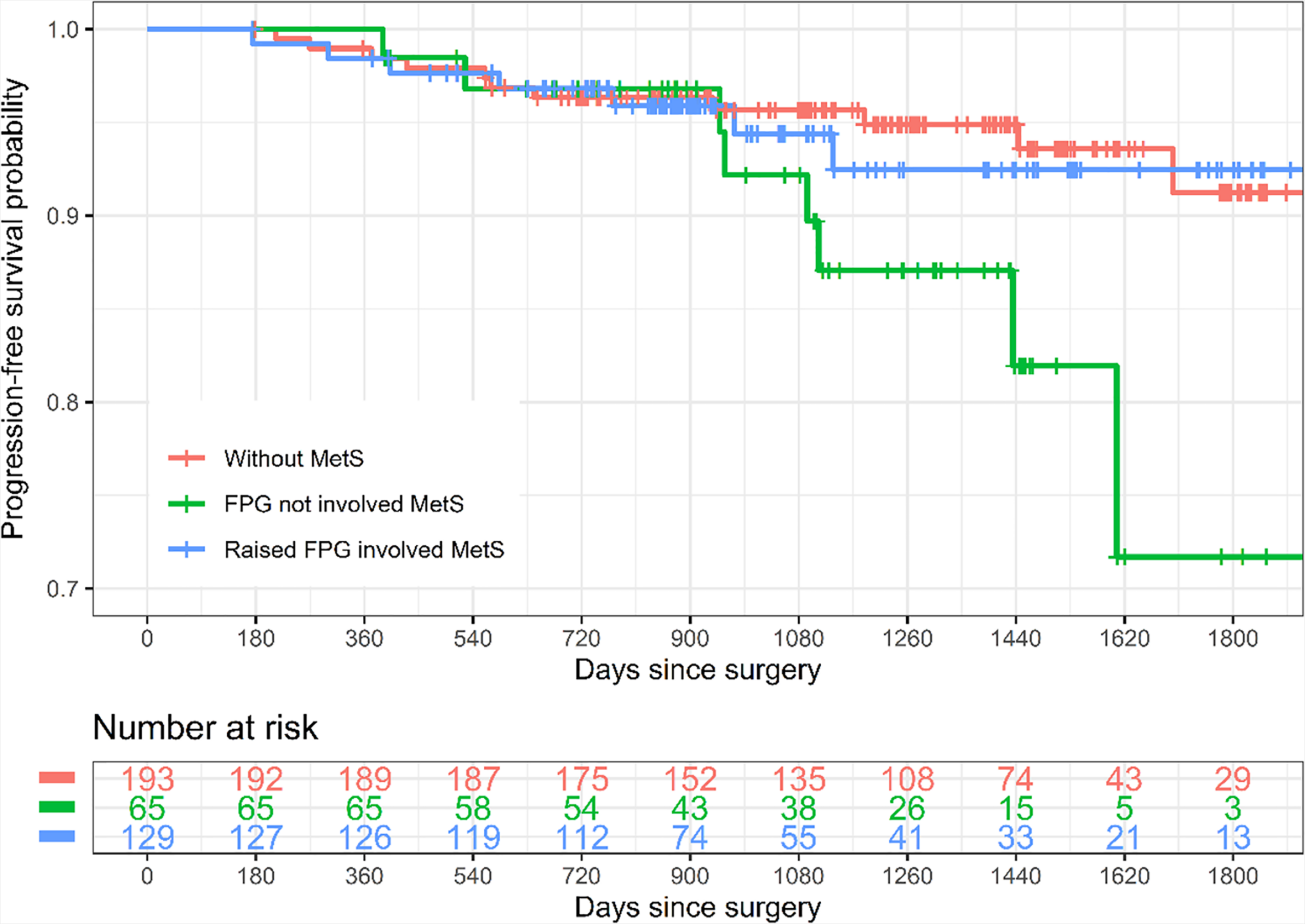
Kaplan–Meier curves of progression-free survival by subtypes of metabolic syndrome. Raised FPG involved MetS refers to MetS diagnosed according to the new International Diabetes Federation (IDF) definition and the patients were with the factor “raised FPG” which were determined by FPG ≥5.6 mmol/l and/or HbA1c ≥6.5%. The rest of the MetS patients were categorized as FPG not involving MetS. MetS, metabolic syndrome; FPG, fasting plasma glucose.

With a median follow-up of 1,253 days, the 5-year cumulative incidence of cancer recurrence was 8.76% (95% confidence interval (CI) 2.5%–14.62%), 28.31% (95% CI 2.33%–47.38%), and 7.54% (95% CI 1.54%–13.17%), respectively (**Table 4** and **Figure 2**). For all-cause mortality, the cumulative incidence was 6.19% (95% CI 1.24%–10.89%), 16.05% (95% CI 0%–34.24%), and 3.91% (95% CI 0%–8.79%), respectively (**Table 4** and **Supplemental Figure 3**).

**Table 4:** Associations of subtypes of metabolic syndrome with prognosis.

	No. at risk	No. events	5-year cumulative incidence (%, 95% CI)	Hazard ratio (95% CI)	
				Crude	Adjusted^†^
Recurrence					
Without MetS	193	11	8.76 (2.5-14.62)	1 (Reference)	1 (Reference)
FPG not involving MetS^‡^	65	8	28.31 (2.33-47.38)	2.59 (1.04-6.48)	2.82 (1.10-7.24)
Raised FPG involving MetS^‡^	129	7	7.54 (1.54-13.17)	1.17 (0.45-3.04)	1.18 (0.45-3.13)
All-cause mortality					
Without MetS	193	8	6.19 (1.24-10.89)	1 (Reference)	1 (Reference)
FPG not involved MetS^‡^	65	3	16.05 (0-34.24)	1.29 (0.34-4.86)	1.30 (0.32-5.27)
Raised FPG involved MetS^‡^	129	3	3.91 (0-8.79)	0.74 (0.20-2.81)	0.83 (0.21-3.25)

†Adjusted for age, menopause, histological grade, tumor size, lymph-vascular space invasion, deep myometrial invasion, type of surgery (Piver–Rutledge classification), adjuvant chemotherapy, and adjuvant radiotherapy.

‡Raised FPG involving MetS refers to MetS diagnosed according to the new International Diabetes Federation (IDF) definition and the patients were with the factor “raised FPG” which were determined by FPG ≥5.6 mmol/l and/or HbA1c ≥6.5%. The rest of the MetS patients were categorized as FPG not involved MetS.

CI, confidence interval; MetS, metabolic syndrome; FPG, fasting plasma glucose.

Crude Cox regression analysis showed that FPG not involving MetS (hazard ratio (HR) 2.59, 95% CI 1.04–6.48) and raised FPG involving MetS (HR 1.17, 95% CI 0.45–3.04, although not statistically significant) were both associated with a higher risk of cancer recurrence when compared to those without MetS, and the association was consistent after adjusting for age, menopause, histological grade, tumor size, lymph-vascular space invasion, deep myometrial invasion, adjuvant chemotherapy, and adjuvant radiotherapy (HR 2.82 (95% CI 1.10–7.24) and 1.18 (95% CI 0.45–3.13), respectively). For the outcome all-cause mortality, although none of the associations were statistically significant, the HRs of FPG not involving MetS were still larger (>1) than that of raised FPG involving MetS when compared to patients without MetS (**Table 4**).

## Discussion

In the current study, we investigated the association between subtypes of MetS and prognosis in patients with stage I endometrioid adenocarcinoma after surgical treatment. Specifically, we used the IDF definition of MetS and categorized subtypes of MetS according to whether raised FPG (or previously diagnosed diabetes) was involved. With a sample size of 387, we found that (1) the prevalence of MetS was about 50% in patients with stage I endometrioid adenocarcinoma; (2) some patterns of MetS were observed, in which reduced HDL cholesterol and raised blood pressure were more prevalent than the other two factors of MetS, and the factor raised FPG was involved in about two-thirds of patients with MetS; (3) comorbid FPG not involving MetS appeared to identify a subgroup of patients with stage I endometrioid adenocarcinoma who had worse prognosis (mainly evaluated by cancer recurrence), and the association remains statistically significant after adjusting for several known prognostic factors. These findings increased the understanding about the association between comorbid MetS and prognosis of patients with stage I endometrioid adenocarcinoma after surgical treatment, and the subtype of MetS we investigated (i.e., according to whether the factor raised FPG was involved) may be helpful to be used as a prognostic predictor. Given the different prognoses but rather similar cancer characteristics between patients with two subtypes of MetS, the findings also indicated that there might be different but not yet identified mechanisms behind and further investigations are warranted. The relatively high prevalence of MetS and relevant cardiovascular risk factors in the patient population also suggest that corresponding management should be considered in addition to cancer treatment.

Our findings are similar to studies that investigated the impact of MetS on prognosis of endometrial cancer. Ni et al. (19) included a total of 385 patients with endometrial adenocarcinoma and compared the survival between patients without and with MetS. The study reported a prevalence of MetS of 33.5% (129/385), and the overall survival rate of patients with MetS was 74.42% compared to 87.89% of patients without MetS. Compared to our study, this study did not only include stage I patients, and the study period was between 2001 and 2008, which might explain why they reported a much lower survival rate. The definition of MetS used in this study appeared to be similar to the IDF definition, but details were limited in the report, and therefore the prevalence of subtypes of MetS cannot be further compared with our studies. Jin et al. (20) used the SEER-Medicare linked database to include 10,090 patients with endometrial cancer (86.6% were stages I to II, between 1992 and 2011) and investigated the difference in cancer-specific survival between comorbid MetS. They reported a MetS prevalence of 16%, and comorbid MetS was associated with worse cancer-specific survival only in early-staged patients. MetS was determined directly by medical records 1 year before cancer diagnosis, which might be at high risk of misclassification and resulted in a low prevalence. Both of these two studies did not investigate subtypes of MetS, but a recent study by Yang et al. (21) did so. In the study by Yang et al. (21), 506 patients with endometrial cancer diagnosed between 2010 and 2016 were included, of which about 70% were stage I and the prevalence of reduced HDL cholesterol and hypertension was lower than our study population. They found that 30% patients were with MetS, and patients with MetS had poor overall survival and recurrence-free survival, although after adjusting for other variables including age, histological type, tumor grade, and stage, MetS was not associated with prognosis. This is somewhat similar to our finding that the association between raised FPG involving MetS was not statistically significantly associated with survival. Yang et al. (21) also found that the prevalence of individual MetS components increased with worse outcomes, while we did not investigate so in the study, as such an investigation might not be very informative. A main difference between the study by Yang et al. (21) and ours is that the definition of MetS used by Yang et al. (21) was based on the 2004 Chinese Diabetes Society standard (i.e., ≥3 the below factors, BMI ≥25 kg/m^2^, hyperglycemia, hypertension, and dyslipidemia).

Many potential mechanisms have been identified about the role of obesity and diabetes (or hyperglycemia) in the development of endometrial cancer (27), including adipocyte-derived estrogen signaling, insulin resistance, and synergistic interaction of estradiol and insulin signaling, while studies on the link between hypertension or lipid disorders and endometrial cancer were relatively limited. Although these studies usually focused on the mechanisms of cancer occurrence but not prognosis of cancer, it is very likely these risk factors (which consist of a MetS diagnosis) would still play a role after endometrial cancer has been developed. The subtypes of MetS we investigated (i.e., whether raised FPG was involved) might represent different molecular mechanisms, and therefore further investigations are warranted. Although there is an argument that clinicians should evaluate and treat all cardiovascular risk factors without regard to whether a patient meets the criteria for diagnosis of MetS (11), to identify a specific combination of cardiovascular risk factors actually did provide relevant prognostic information as seen in our study, compared to evaluating cardiovascular risk factors individually (28). The different distribution patterns of cardiovascular risk factors in patients with MetS also suggest that there might be interactions between these cardiovascular risk factors, which would not be observed if they were studied individually.

There are some limitations in our study. First, we did not have information of waist circumference to define central obesity, and we used data from a Chinese female population to develop a prediction model to estimate waist circumference by age and BMI. This obviously introduces risk of misclassification of central obesity and MetS. However, there is no reason to suspect that the direction of misclassification would only be toward one direction. Among those who were identified as MetS in our study, the mean BMI was 26.7 ± 3.2 kg/m^2^, and 77.8% and 24.2% of them met the criteria of overweigh (BMI ≥24 kg/m^2^) and obesity (BMI ≥28 kg/m^2^), respectively (data were not shown in the manuscript), which somewhat relaxed the concern, although obesity defined by BMI does not equal to central obesity. Another support came from a recent investigation on prevalence of central obesity in a Chinese population (29), which was found for women with a BMI of 24.0–27.9 kg/m^2^, and the prevalence of central obesity (defined as waist circumference ≥85cm) was 47.3% (45.5%–49.2%). In our study population, when using a cutoff of 85 cm to define central obesity, the prevalence was 47.8% (data were not shown in the manuscript), which was rather consistent with this report. However, studies are needed to confirm our findings. Second, there are other versions of MetS diagnosis criteria, so it remains unknown whether using other MetS diagnosis criteria will draw the same conclusion. Third, residual confounding is still possible, although we already adjusted many known prognostic risk factors. Fourth, the sample size and lengths of follow-up in our study are still relatively limited, especially considering that the recurrence rate is relatively low in absolute scale within a short follow-up period, so studies with larger sample sizes and longer follow-up are encouraged to confirm our findings. Last but not least, it remains unclear whether the association between FPG not involving MetS and much worse prognosis is causal, and whether intervention of dyslipidemia would benefit survival.

## Conclusion

Comorbid MetS generally presents as a risk factor of poor prognosis in patients with stage I endometrioid adenocarcinoma after surgical treatment, but the magnitude of the association may vary between subtypes, in which FPG not involving MetS appears to be predominant.

## Data availability statement

The original contributions presented in the study are included in the article/**Supplementary Material**. Further inquiries can be directed to the corresponding authors.

## Ethics statement

This study was reviewed and approved by Sun Yat-sen Memorial Hospital (No. SYSEC-KY-2022-125). The patients/participants provided their written informed consent to participate in this study.

## Author contributions

M-QC: Data curation, formal analysis, methodology, writing original draft and project administration. H-XL: Data curation, methodology, formal analysis, writing original draft and project administration. J-XL: Methodology, formal analysis and data curation. M-FW: Methodology, data curation. JL: Conceptualization, methodology, supervision, and writing–review and editing. L-JW: Conceptualization, supervision, resources, and writing–review and editing. All authors contributed to the article and approved the submitted version.

## Conflict of interest

The authors declare that the research was conducted in the absence of any commercial or financial relationships that could be construed as a potential conflict of interest.

## Publisher’s note

All claims expressed in this article are solely those of the authors and do not necessarily represent those of their affiliated organizations, or those of the publisher, the editors and the reviewers. Any product that may be evaluated in this article, or claim that may be made by its manufacturer, is not guaranteed or endorsed by the publisher.

## Supplementary material

Supplementary Figure 1 Distributions and correlations of age, BMI, and waist circumference of the data used for developing the model for predicting waist circumference by age and BMI. The dataset used was accessed from: Heil, Daniel; Zhu, Wei (2018), “Data for: Associations of Vitamin D Status with Markers of Metabolic Health: A Community-Based Study in Shanghai, China”, Mendeley Data, V1, doi: 10.17632/h475rmyd5f.1, which is licensed under a Creative Commons Attribution 4.0 International license. Only data of female individuals without missing values of age, BMI, and waist circumferences were used in this study. Correlations were evaluated by Pearson correlation coefficient, and *** indicates P < 0.001. Abbreviation: BMI, body mass index.

Supplementary Figure 2 The original waist circumference versus the predicted values by the developed model in the data used for developing the model for predicting waist circumference by age and BMI. Notes: The dataset used was accessed from: Heil, Daniel; Zhu, Wei (2018), “Data for: Associations of Vitamin D Status with Markers of Metabolic Health: A Community-Based Study in Shanghai, China”, Mendeley Data, V1, doi: 10.17632/h475rmyd5f.1, which is licensed under a Creative Commons Attribution 4.0 International license. Only data of female individuals without missing values of age, BMI, and waist circumferences were used in this study. In the figure, the original values of waist circumference are presented in black, and the predicted values are presented in red, using the prediction model: Waist circumference (cm) = 2.20730 × BMI (kg/m2) + 0.16377 × Age (years) + 22.23815. BMI, body mass index.

Supplementary Figure 3 Kaplan-Meier curves of overall survival by subtypes of metabolic syndrome. Raised FPG involved MetS refers to MetS diagnosed according to the new International Diabetes Federation (IDF) definition and the patients were with the factor “raised FPG” which were determined by FPG ≥5.6 mmol/L and/or HbA1c ≥6.5%. The rest of the MetS patients were categorized as FPG not involved MetS. MetS, metabolic syndrome; FPG, fasting plasma glucose.
