# Supplementary material for: Association between subtypes of metabolic syndrome and prognosis in patients with stage I endometrioid adenocarcinoma: A retrospective cohort study
Source: Front Oncol. 2022 Sep 20;12:950589. doi: 10.3389/fonc.2022.950589 (PMC9530564; doi:10.3389/fonc.2022.950589)
Supplement: Supplementary file 1 [file Table_1.docx]

**Supplemental Table 1 Personal characteristics of the data used for developing the model for predicting waist circumference by age and BMI**

|  | N | Mean±SD | Median (25^th^-75^th^ percentile) | Min-Max |
| --- | --- | --- | --- | --- |
| Age (years) | 312 | 51.3±12.1 | 54 (44.25-60) | 18-74 |
| BMI (kg/m^2^) | 312 | 23.5±3.1 | 23.3 (21.4-25.2) | 15.7-36.4 |
| Waist circumference (cm) | 312 | 82.4±9.2 | 83 (76-88) | 60-114 |

Note: The dataset used was accessed from: Heil, Daniel; Zhu, Wei (2018), “Data for: Associations of Vitamin D Status with Markers of Metabolic Health: A Community-Based Study in Shanghai, China”, Mendeley Data, V1, doi: 10.17632/h475rmyd5f.1, which is licensed under a Creative Commons Attribution 4.0 International license. Only data of female individuals without missing values of age, BMI, and waist circumferences were used in this study.

Abbreviations: BMI, body mass index; SD, standard deviation.
