# Supplementary material for: Association between subtypes of metabolic syndrome and prognosis in patients with stage I endometrioid adenocarcinoma: A retrospective cohort study
Source: Front Oncol. 2022 Sep 20;12:950589. doi: 10.3389/fonc.2022.950589 (PMC9530564; doi:10.3389/fonc.2022.950589)
Supplement: Supplementary file 3 [file Table_3.docx]

**Supplemental Table 3 Distribution of the four factors in the patients who were diagnosed as metabolic syndrome**

|  | TG | HDL | BP | FPG |
| --- | --- | --- | --- | --- |
| TG | 115 (100%) | 97 (58.8%) | 89 (57.1%) | 80 (62.0%) |
| HDL | 97 (84.3%) | 165 (100%) | 130 (83.3%) | 105 (81.4%) |
| BP | 89 (77.4%) | 130 (78.8%) | 156 (100%) | 98 (76.0%) |
| FPG | 80 (69.6%) | 105 (63.6%) | 98 (62.8%) | 129 (100%) |

Note: TG, HDL, BP, FPG refer to the four factors for diagnosing metabolic syndrome, namely raised triglycerides, reduced HDL cholesterol, raised blood pressure, and raised fasting plasma glucose, respectively. All the patients were with central obesity.

Abbreviations: TG, triglycerides; HDL, high-density lipoprotein; BP, blood pressure; FPG, fasting plasma glucose.
