# Supplementary figures and images for: Association between subtypes of metabolic syndrome and prognosis in patients with stage I endometrioid adenocarcinoma: A retrospective cohort study
Source: Front Oncol. 2022 Sep 20;12:950589. doi: 10.3389/fonc.2022.950589 (PMC9530564; doi:10.3389/fonc.2022.950589)

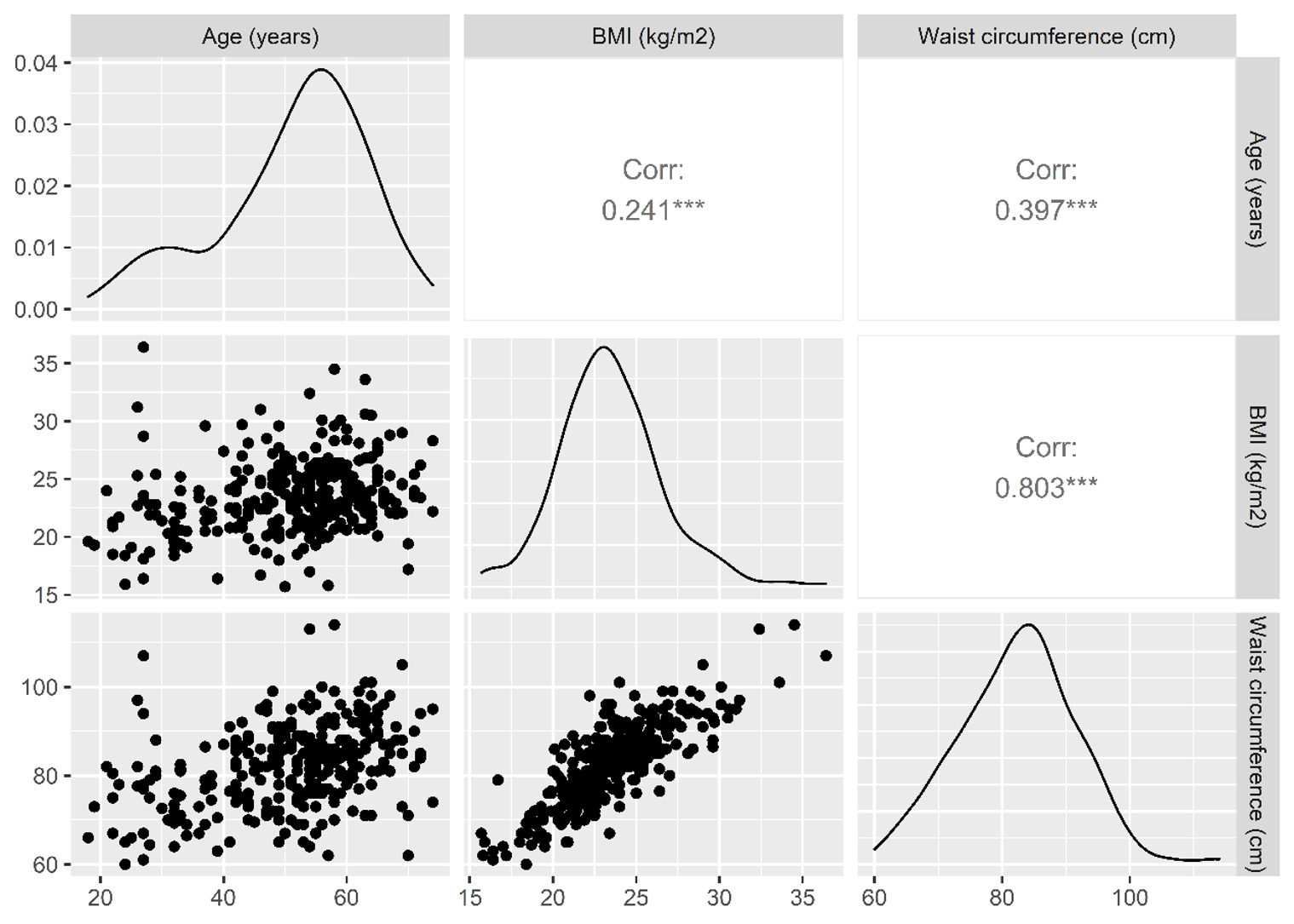

Supplement: Supplementary Figure 1 — Distributions and correlations of age, BMI, and waist circumference of the data used for developing the model for predicting waist circumference by age and BMI. The dataset used was accessed from: Heil, Daniel; Zhu, Wei (2018), “Data for: Associations of Vitamin D Status with Markers of Metabolic Health: A Community-Based Study in Shanghai, China”, Mendeley Data, V1, doi: 10.17632/h475rmyd5f.1, which is licensed under a Creative Commons Attribution 4.0 International license. Only data of female individuals without missing values of age, BMI, and waist circumferences were used in this study. Correlations were evaluated by Pearson correlation coefficient, and *** indicates P < 0.001. Abbreviation: BMI, body mass index. [file Image_1.tif]

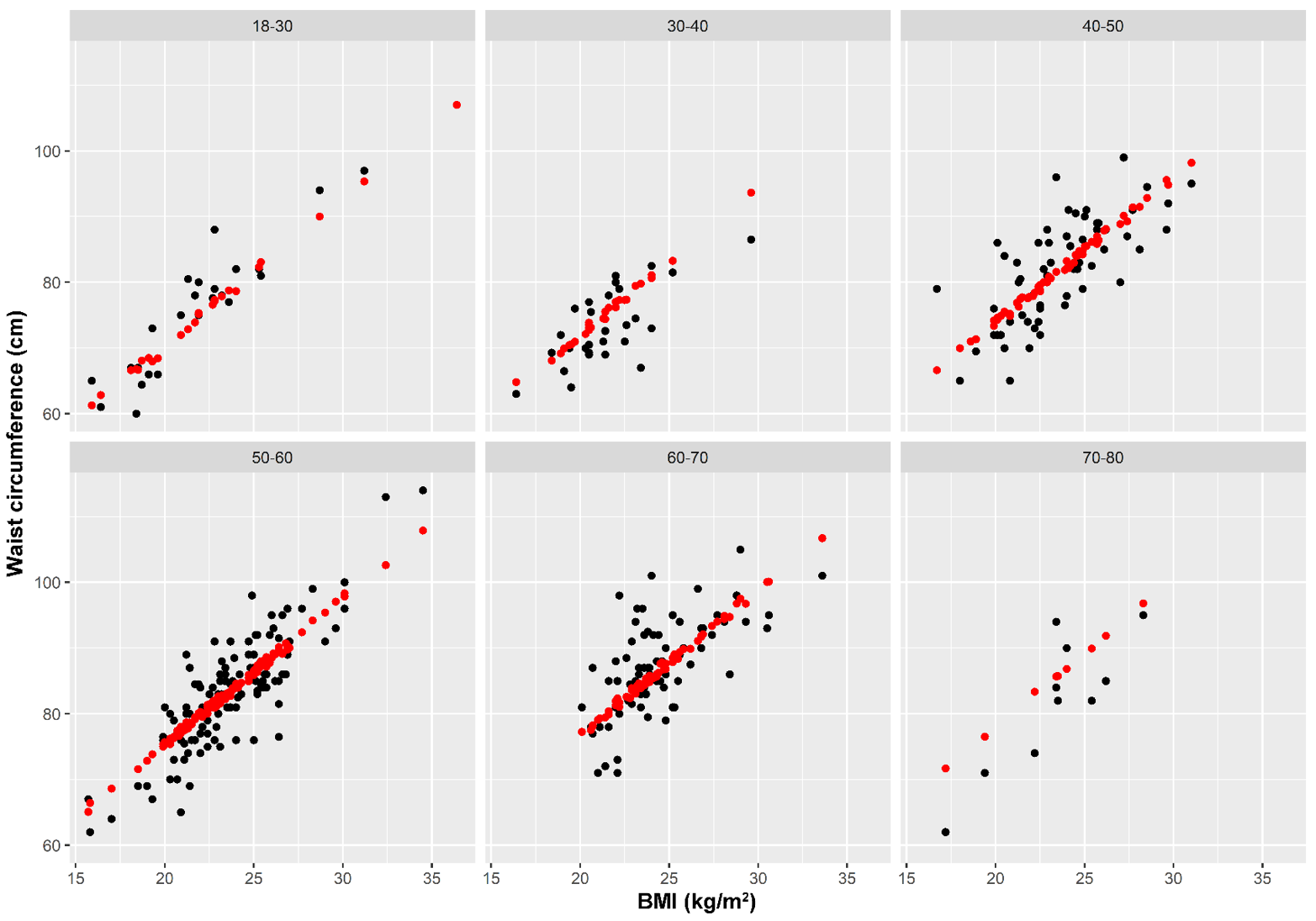

Supplement: Supplementary Figure 2 — The original waist circumference versus the predicted values by the developed model in the data used for developing the model for predicting waist circumference by age and BMI. Notes: The dataset used was accessed from: Heil, Daniel; Zhu, Wei (2018), “Data for: Associations of Vitamin D Status with Markers of Metabolic Health: A Community-Based Study in Shanghai, China”, Mendeley Data, V1, doi: 10.17632/h475rmyd5f.1, which is licensed under a Creative Commons Attribution 4.0 International license. Only data of female individuals without missing values of age, BMI, and waist circumferences were used in this study. In the figure, the original values of waist circumference are presented in black, and the predicted values are presented in red, using the prediction model: Waist circumference (cm) = 2.20730 × BMI (kg/m2) + 0.16377 × Age (years) + 22.23815. BMI, body mass index. [file Image_2.tif]

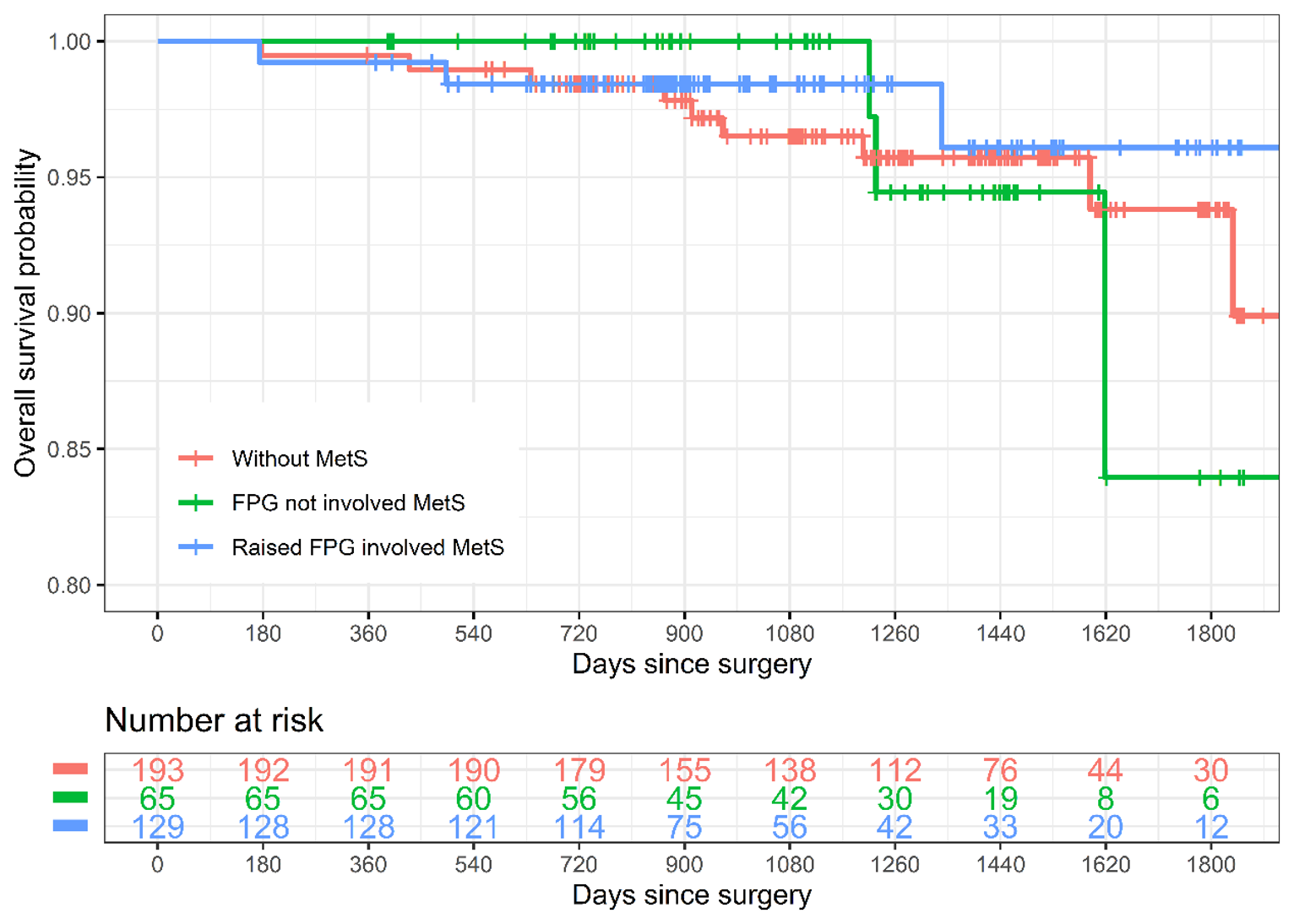

Supplement: Supplementary Figure 3 — Kaplan-Meier curves of overall survival by subtypes of metabolic syndrome. Raised FPG involved MetS refers to MetS diagnosed according to the new International Diabetes Federation (IDF) definition and the patients were with the factor “raised FPG” which were determined by FPG ≥5.6 mmol/L and/or HbA1c ≥6.5%. The rest of the MetS patients were categorized as FPG not involved MetS. MetS, metabolic syndrome; FPG, fasting plasma glucose. [file Image_3.tif]
